# Multi-Focus Color Image Fusion Based on Quaternion Multi-Scale Singular Value Decomposition

**DOI:** 10.3389/fnbot.2021.695960

**Published:** 2021-06-23

**Authors:** Hui Wan, Xianlun Tang, Zhiqin Zhu, Bin Xiao, Weisheng Li

**Affiliations:** ^1^College of Computer Science and Technology, Chongqing University of Posts and Telecommunications, Chongqing, China; ^2^College of Computer and Information Science, Chongqing Normal University, Chongqing, China; ^3^College of Automation, Chongqing University of Posts and Telecommunications, Chongqing, China

**Keywords:** multi-focus color image, image fusion, quaternion, singular value decomposition, multi-scale decomposition

## Abstract

Most existing multi-focus color image fusion methods based on multi-scale decomposition consider three color components separately during fusion, which leads to inherent color structures change, and causes tonal distortion and blur in the fusion results. In order to address these problems, a novel fusion algorithm based on the quaternion multi-scale singular value decomposition (QMSVD) is proposed in this paper. First, the multi-focus color images, which represented by quaternion, to be fused is decomposed by multichannel QMSVD, and the low-frequency sub-image represented by one channel and high-frequency sub-image represented by multiple channels are obtained. Second, the activity level and matching level are exploited in the focus decision mapping of the low-frequency sub-image fusion, with the former calculated by using local window energy and the latter measured by the color difference between color pixels expressed by a quaternion. Third, the fusion results of low-frequency coefficients are incorporated into the fusion of high-frequency sub-images, and a local contrast fusion rule based on the integration of high-frequency and low-frequency regions is proposed. Finally, the fused images are reconstructed employing inverse transform of the QMSVD. Simulation results show that image fusion using this method achieves great overall visual effects, with high resolution images, rich colors, and low information loss.

## Introduction

Image fusion is the process of combining the information from two or more images into a single image. It has been applied widely, ranging from medical analysis (Jin et al., 2018a,b, 2020), to remote sensing imaging and artificial fog removal (Zhu et al., 2020). An important branch of image fusion is multi-focus image fusion, which integrates images with different focal points into a full-focus image with global clarity and rich details. Multi-focus image fusion algorithms mainly include spatial domain methods, transform domain methods, and deep learning methods (Liu S. et al., 2020; Liu Y. et al., 2020).

The spatial domain methods can be grouped into pixel-based method, block-based method, and region-based method (Jin et al., 2018a,b; Qiu et al., 2019; Xiao et al., 2020). Compared with the pixel-based method, the other two use the spatial correlation of adjacent pixels to guide image fusion to avoid contrast reducing and detail loss in the fusion images. First, the original images are divided into a number of blocks or regions, and then the focus level and sharpness of each block or region is measured by image intensity information. Finally, a block or region with a higher degree of focus as part of the fusion image is selected. Vishal and Vinay (2018) proposed a block-based spatial domain multi-focus image fusion method, and used spatial frequency to measure the focus level of the blocks. Duan et al. (2018) proposed a segmentation scheme based on enhanced LSC, which embeds the depth information of pixels in the clustering algorithm for multi-focus image fusion. The main advantage of fusion methods based on spatial domain lies in the fact that simple to implement, it can obtain the focus measure with low computational complexity. However, the quality of image fusion is relevant to the selection of image block sizes or the segmentation algorithms. When the size of the image block is not properly selected, the fusion image may generate a “block effect.” And if the segmentation algorithm fails to segment the region accurately, the focused region cannot be determined and extracted correctly.

In the transform domain approach, various multi-scale decomposition (MSD) methods are applied to multi-focus image fusion. Multi-scale decomposition algorithm mainly includes pyramid transform (Burt and Kolczynski, 1993; Du et al., 2016), wavelet transform (Gonzalo and Jesús, 2002; Jaroslav et al., 2002) and multi-scale geometric analysis (Li et al., 2017, 2018; Liu et al., 2019a). Compared with the pyramid and wavelet transforms, though the multi-scale geometric analysis method outperforms the pyramid and wavelet transforms in feature representation and excels in capturing multi-directional information and translation invariance, it is not time-efficient when it comes to decomposition and reconstruction. In addition to traditional multi-scale decomposition methods mentioned above, some other multi-scale fusion methods have been proposed. Zhou et al. (2014) proposed a novel image fusion scheme based on large and small dual-scale decomposition. In this scheme, the two-scale method is used to determine the image gradient weight, and removes the influence of anisotropic blur on the focused region detection effectively. An and Li (2019) introduced a novel adaptive image decomposition algorithm into the field of image processing, which can fast decompose images and has multi-scale characteristics. Zhang et al. (2017) proposed a multi-scale decomposition scheme by changing the size of the structural elements, and extracting the morphological gradient information of the image on different scales to achieve multi-focus image fusion. Ma et al. (2019) proposed a multi-focus image fusion method based on to estimate a focus map directly using small-scale and large-scale focus measures. Naidu (2011) proposed a novel method of multi-focus images fusion. In this method, multi-scale analysis and singular value decomposition are combined to perform multi-scale singular value decomposition on multi-focus images to obtain low-frequency sub-images and high-frequency sub-images of different scales. This multi-scale decomposition method has the stability and orthogonality of SVD. Since no convolution operation is required, the decomposition speed is fast.

Deep learning methods, which can be further grouped into classification model based methods and regression model based methods (Liu Y. et al., 2020). In the classification model, Liu et al. (2017) first introduced convolutional neural networks (CNN) into the field of multi-focus image fusion. With this method, the activity level measurement and the fusion rule can be jointly generated by learning a CNN model. In the regression model, Li et al. (2020) proposed a novel deep regression pair learning convolutional neural network for multi-focus image fusion. This method directly converts the entire image into a binary mask as the input of the network without dividing the input image into small patche, thereby solving the problem of the blur level estimation around the focused boundary due to patche division. These methods can extract more image features through self-learning of the deep network, and carry out image fusion based on these features. However, the difficulties in training a large number of parameters and large datasets have directly affected the image fusion efficiency and quality. Compared with deep learning methods, the conventional fusion methods are more extensible and repeatable, facilitating real-world applications. Thus, this paper mainly aims to improve the conventional multi-focus image fusion algorithms.

Most of the existing multi-focus image fusion algorithms mentioned above can process gray and color multi-focus images. As for the color multi-focus image fusion, each color channel is fused separately, and then combined to get the final fused image (Naidu, 2011; Liang and He, 2012; Aymaz and Köse, 2019). These traditional fusion methods ignore the inter-relationship between the color channels, which will lead to hue distortions and blur in the image fusion process. To solve the above problems, this paper proposes a novel mathematical model for color images based on quaternion matrix analysis. This model considers the human visual characteristics and interaction between pixels in color images and combines quaternion with multi-scale singular value decomposition (MSVD) (Kakarla and Ogunbona, 2001; Naidu, 2011). In this method, the three color components of a color image are decomposed as a whole to extract the rich color and detail information. Firstly, the three color components of the pixel are represented by three imaginary parts of a quaternion. Secondly, the multi-focus color image represented by the quaternion matrix is decomposed into a low-frequency sub-image and several high-frequency sub-images using multi-scale singular value decomposition (MSVD). The former contains the approximate structure and color information of the source image, the latter contains detailed features. Then, the low-frequency component and the high-frequency component are respectively fused based on different fusion rules. The designed fusion rule makes full use of the decomposition coefficient represented by the quaternion and applies the structural information and color information of the image to the fusion. Finally, the fusion components are used to reconstruct the fusion image. The fused image can more accurately maintain the spectral characteristics of the color channel. We define this method as quaternion multi-scale singular value decomposition (QMSVD). The main innovations of this method are listed below:

- The combination of quaternion and multi-scale singular value decomposition is applied to multi-focus color image fusion for the first time. That is, the color image represented by the quaternion is decomposed by multi-scale singular value decomposition, and the sub-images obtained by decomposition better retain the structure and color information of the original image.
- The multi-channel is introduced into the QMSVD for the first time, and achieve the purpose of extracting the salient features on the channels of different decomposition layers for image fusion.
- In the fusion of low-frequency sub-images, in order to make full use of the color information of the image, an improved fusion rule of local energy maximization is proposed, and the fusion rule introduces the color difference between pixels and combines local energy. In the fusion of high-frequency sub-images, the fusion results of low-frequency coefficients are incorporated into the fusion of high-frequency sub-images, and a local contrast fusion rule based on the integration of high-frequency and low-frequency regions is proposed.

The structure of this paper is organized as follows. Section Multi-Scale Singular Value Decomposition of a Color Image introduces the concept of multi-scale singular value decomposition of a color image. Section Multi-Focus Color Image Fusion Based on QMSVD proposes multi-focus color image fusion model based on QMSVD. Section Experimental Results and Discussion we compare and analyze the results obtained through the state-of-the-art methods. Finally, conclusions for this paper are made in section Conclusion.

## Multi-Scale Singular Value Decomposition of a Color Image

To decompose the color image we integrate quaternion representation of color image with multi-scale decomposition. In this way, the approximate and detailed parts represented by quaternion can be obtained. The two parts are respectively fused, and the fused components are used to reconstruct the fusion image.

### Quaternion Representation of a Color Image

Quaternions were discovered in 1843 by the Irish mathematician and physicist William Rowan Hamilton. It is extension of ordinary complex number, which extends ordinary complex numbers from a two-dimensional space to a four-dimensional space. A quaternion is composed of a real part and three imaginary parts. The operations of the three imaginary parts are equivalent, which makes it very suitable for describing color images and expressing the internal connection of color channels. The three color channels of the image can be represented by three imaginary parts of quaternion (Chen et al., 2014; Xu et al., 2015; Grigoryan and Agaian, 2018). The general form of a quaternion is *q* = *q*_*a*_ + *q*_*b*_*i* + *q*_*c*_*j* + *q*_*d*_*k*. It contains one real part *q*_*a*_ and three imaginary parts *q*_*b*_*i*, *q*_*c*_*j* and *q*_*c*_*k*, if the real part *q*_*a*_ of a quaternion *q* is zero, *q* is called a pure quaternion. The conjugation of quaternions is defined as:

(1) $$\begin{array}{l} q^{*}=q_{a}-q_{b}i-q_{c}j-q_{d}k \end{array}$$

The modulus of a quaternion is defined as:

(2) $$\begin{array}{l} |q|=\sqrt{qq^{*}}=\sqrt{q_{a}^{2}+q_{b}^{2}+q_{c}^{2}+q_{d}^{2}} \end{array}$$

The rotation theory of quaternions is stated as follows:

In the three-dimensional space, *u* is a unit of pure quaternion, and the modulus is |*u*| = 1. If *R* = *e*^*uθ*^, then RXR^*^ indicates that the pure quaternion *X* is rotated by 2θ radians about the axis. *u* and θ are defined as:

$$\begin{array}{lll} u & = & \frac{1}{\sqrt{q_{b}^{2}+q_{c}^{2}+q_{d}^{2}}}\left(q_{b}i+q_{c}j+q_{b}iq_{d}k\right)\\ \theta & = & \{\begin{array}{cc} {\text{tan}}^{-1}\sqrt{q_{b}^{2}+q_{c}^{2}+q_{d}^{2}}/q_{a}, & q_{a}\neq 0\\ \pi /2 & q_{a}=0 \end{array} \end{array}$$

Let $u=\left(i+j+k\right)/\sqrt{3}$, which represents a three-dimensional grayscale line in RGB space. The three color components of the pixels on the grayscale line are all equal. Let θ = π/2, that is:

(3) $$\begin{array}{l} RXR^{*}=e^{u\pi /2}X{\left(e^{u\pi /2}\right)}^{*}=\left(i+j+k\right)/\sqrt{3}\text{*}X\text{*}\left(-i-j-k\right)/\sqrt{3} \end{array}$$

Equation (3) means that *X* is rotated around the gray line *u* by 180 degree. That is, *X* is turned to the opposite direction with *u* as the axis of symmetry. Then, the pixel *X* + RXR^*^ falls on the grayscale line.

A color image can be represented as a pure quaternion, that is:

(4) $$\begin{array}{l} f\left(x,y\right)=f_{R}\left(x,y\right)\cdot i+f_{G}\left(x,y\right)\cdot j+f_{B}\left(x,y\right)\cdot k \end{array}$$

In Equation (4), *f*_*R*_(*x, y*), *f*_*G*_(*x, y*), *f*_*B*_(*x, y*) represent the R, G, and B color channel components of the color image, respectively. The *x, y* represent the rows and columns of the color image matrix, where the pixels reside. Such a color image can be represented by a quaternion matrix, and the processing of the color image can be performed directly on the quaternion matrix. In contrast with the traditional approaches, which convert a color image to a grayscale one or process each color channel separately, the quaternion method can process the color image as a whole.

### Multi-Scale Decomposition of a Color Image

The singular value decomposition is an important matrix decomposition in linear algebra (Liu et al., 2019b), and it is to decompose the image matrix diagonally according to the size of the eigenvalues. There is no redundancy among the decomposed images, and it is suitable to use different fusion rules for the fusion of each sub-image. We extend decomposition to the multi-scale form in this section. Using multi-scale can perform image fusion in different scales and different directions.

*X*_*q*_ is the quaternion matrix form of the color image *f*(*x, y*). The rank of the *m* × *n* quaternion matrix *X*_*q*_ is *r*. Given the *m*×*m* quaternion unitary matrix *U*_*q*_ and *n*×*n* quaternion unitary matrix *V*_*q*_, we can get:

(5) $$\begin{array}{l} {\left(U_{q}\right)}^{H}X_{q}V_{q}=\left[\begin{array}{cc} \Lambda_{r} & 0\\ 0 & 0 \end{array}\right]\equiv \Lambda \in R^{m\times n} \end{array}$$

where the superscript *H* represents conjugate transpose, and Λ_*r*_ = *diag*{λ_1_, λ_2_, ⋯ , λ_*r*_}, λ_*i*_(1 ≤ *i* ≤ *r*) is the singular value of *X*_*q*_, λ_1_ ≥ λ_2_ ≥ ⋯ ≥ λ_*r*_. It follows that the singular value decomposition of the quaternion matrix *X*_*q*_ is:

(6) $$\begin{array}{l} X_{q}=U_{q}\left[\begin{array}{cc} \Lambda_{r} & 0\\ 0 & 0 \end{array}\right]{\left(V_{q}\right)}^{H} \end{array}$$

In Equation (6), $U_{q}{\left(U_{q}\right)}^{H}=I^{m\times m},V_{q}{\left(V_{q}\right)}^{H}=I^{n\times n}\text{Unit matrix}$.

The multi-scale singular value decomposition of a color image represented by a quaternion can be realized, according to the ideas proposed in Naidu (2011). The *M* × *N* color image *X*_*q*_, represented by the quaternion, is divided into non-overlapping *m* × *n* blocks, and each sub-block is arranged into an *mn* × 1 vector. By combining these column vectors, a quaternion matrix ${X_{q}}^{\prime }$ with a size of can be obtained. The singular value decomposition of ${X_{q}}^{\prime }$ is:

(7) $$X_{q}^{\prime }=U_{q}^{\prime }\Lambda^{\prime }{\left(V_{q}^{\prime }\right)}^{H}$$

${U_{q}}^{\prime }$ and ${V_{q}}^{\prime }$ are orthogonal matrices, and Λ′ is a non-singular diagonal matrix after ${X_{q}}^{\prime }$ decomposition. According to Equation (7):

(8) $$S={\left(U_{q}^{\prime }\right)}^{H}X_{q}^{\prime }=\Lambda^{\prime }{\left(V_{q}^{\prime }\right)}^{H}$$

the size of the quaternion matrix *S* is *mn* × *MN*/*mn*.

According to the singular value decomposition mentioned above, the first column vector of ${U_{q}}^{\prime }$ corresponds to the maximum singular value. When it is left multiplied by the matrix ${X_{q}}^{\prime }$, the first row *S*(1, :) of *S* carries the main information from the original image, which can be regarded as the approximate, or smooth component of the original image. Similarly, the other rows *S*(2 : *mn*, :) of *S* correspond to smaller singular values, which retain such detailed information as the texture and edge. Therefore, through singular value decomposition, the image can be decomposed into low-frequency and high-frequency sub-images by the singular value to achieve the multi-scale decomposition of the image. In the QMSVD approach, decomposition is goes layer by layer, repeating the process above. In repeated decomposition, the approximate component *S*(1, :) of the upper layer is used to replace the next layer of *X*_*q*_.

When the original image is divided into *m* × *n* blocks, according to the different values of *m* and *n*, QMSVD can be called (*m* × *n*)-channel QMSVD. For example: when *m* = *2* and *n* = *2*, it is called four-channel QMSVD when *m* = *2* and *n* = *3* or *m* = *3* and *n* = *2*, it is called six-channel QMSVD, when *m* = *2* and *n* = *4* or *m* = *4* and *n* = *2*, it is called eight-channel QMSVD.

We take six-channel QMSVD as an example to illustrate the decomposition structure of each layer. Let *m* = *2, n* = *3*, and *m*×*n* = *6*:

(9) $$\begin{array}{l} \phi_{LL}=S\left(1:\right)\begin{array}{l} \psi_{H1}=S\left(2:\right),\psi_{H2}=S\left(3:\right),\psi_{H3}=S\left(4:\right)\\ \psi_{H4}=S\left(5:\right),\psi_{H5}=S\left(6:\right) \end{array}\\ X_{q}\to \left\{\phi_{LL},\left\{\psi_{H1},\psi_{H2},\psi_{H3},\psi_{H4},\psi_{H5}\right\},U\right\} \end{array}$$

In Equation (9), the lowest-resolution approximation component vector is ϕ_*LL*_, the detail component vectors are {ψ_*H*1_, ψ_*H*2_, ψ_*H*3_, ψ_*H*4_, ψ_*H*5_}, and the eigenvector matrix is *U*. During the transformation of the lower layer, ϕ_*LL*_ is replaced with *X*_*q*_, the decomposition operates by Equation (9) and the next layer decomposition is obtained, and the multilayer decomposition of the image can be obtained by repeating the process. Because the decomposition process is reversible, the original image can be reconstructed by inverse transformation of QMSVD.

The QMSVD method proposed in this paper, the MSVD (Naidu, 2011) method and the QSVD (Bihan and Sangwine, 2003) method all decompose the image through singular value decomposition, but they have their distinct characteristics. In Naidu (2011), the MSVD is mainly a decomposition method for gray images. When decomposing a color image, the MSVD method is used on each color channel, and then combine the three decomposed color channels to obtain a decomposed color image. This decomposition method of channel information separation ignores the correlation between channels and take no account of color information of image. The QMSVD method overcomes the shortcomings of the MSVD method, and can maintain the correlation between color channels while decomposing color images. Compared with the QMSVD method, QSVD directly decomposes color images to get the eigenvalues and corresponding eigenvectors. Then, according to experience, we use the truncation method on QSVD to divide the eigenvalues in a descending order into different segments to realize image decomposition. However, the decomposition process based on experience truncation method lacks a definite physical meaning. In order to ascribe a clear physical and geometric meaning to the decomposition process, the multi-channel QMSVD is introduced, which directly decomposes the image into low-frequency and high-frequency components of different scales according to the size of eigenvalues.

**Figure 1:**
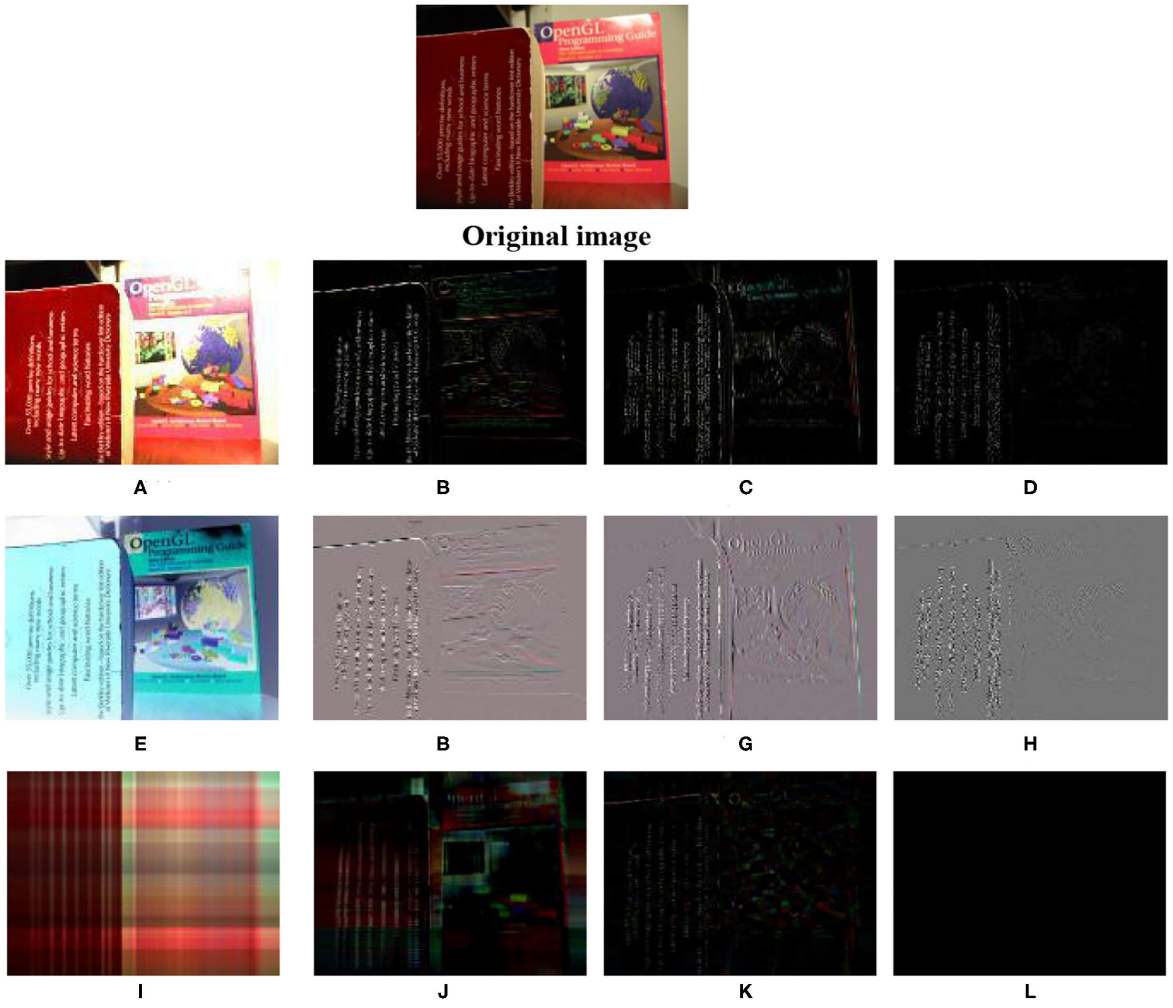
This figure shows the decomposition of color image by QMSVD, MSVD, and QSVD. **(A)** The low-frequency image of the origianl image after decomposed by QMSVD, and **(B–D)** the high-frequency images of the origianl image. **(E)** The low-frequency image of the original image after decomposed by MSVD, and **(F–H)** the high-frequency images of the original image. **(I)** The decomposition image corresponding to the first eigenvalue of the original image decomposed by QSVD, and **(J)** the decomposition image corresponding to the eigenvalue truncated from the 2th to the 25th after QSVD decomposition, **(K)** the decomposition image corresponding to the eigenvalue truncated from the 26th to the 50th, **(L)** the decomposition image corresponding to the eigenvalue truncated from the 51th to the 240th. The eigenvalues are arranged from large to small.

Figure 1 compares the results achieved by three decomposition methods. It can be seen that: (1) The QMSVD method directly decomposes the color image into a low-frequency component and three high-frequency components. The low frequency component is an approximation of the original image, which retains the characteristics of the original image in terms of structure and color. The high-frequency components extract the edge and contour features of the original image. (2) The MSVD method does not directly decompose the color image. First, decompose each color channel, and then combine the decomposed components into low-frequency components and high-frequency components. Compared with the QMSVD method, the low-frequency component does not retain the color characteristics of the original image. As it can be seen from the Figure 1, the main color of the low-frequency component is blue, while the main color of the original image is red. The high-frequency component extracts the edge and contour features of the original image, but does not have the fine features extracted by the QMSVD method. This is due to the fact that the edge features of each component cannot be completely overlapped when the components are combined. (3) Compared with the QMSVD method, the QSVD method is not strong on extracting detailed features. It can be seen from the Figure 1 that the main structure and color information are in the decomposed image corresponding to the first feature value, and the other feature values are truncated into three segments, corresponding to the three decomposed images respectively, and these images only carry a small amount of detailed features. Since the QSVD method is mainly used for image compression, in the experimental comparison part, we only compare QMSVD with MSVD methods.

## Multi-Focus Color Image Fusion Based on QMSVD

### Low-Frequency Component Fusion Rules

The low-frequency sub-image of QMSVD reflects the overall characteristics of the color original image. Commonly used low-frequency sub-image fusion rules include weighted average and maximum local energy. The weighted average rule is to get the fusion coefficient by weighted average of the low frequency coefficients in the same position of the images, which will result in the decline in the contrast of the fused image. The rule of maximum local energy is to compare the energy of low-frequency coefficients at the same position of the images, and choose the higher energy as the fusion coefficient. This fusion rule only considers the local energy of the image, and does not factor in the color information contained in the color image, so the visual effect of the color fusion image is not desirable. In order to overcome the inadequacy, QMSVD uses a quaternion to represent the color image, and calculates the color differences between two color pixels based on the quaternion rotation theory. The coefficient window energy is used as activity level of the low frequency component, and the color difference between the color pixels in the center of the coefficient window is deemed as the matching level, with both jointly participating in the decision mapping.

#### Activity Level

Given the human visual system is sensitive to local variation, local window energy is used as the measurement of activity level. Local areas with larger variance exhibit greater contrast between pixels, and stronger window activity level. In contrast, pixel values more uniform in local areas with smaller variance, display weaker window activity level. Therefore, the pixel with the highest contrast in the low-frequency coefficient is selected as the fusion result.

(10) $$\begin{array}{l} a_{S}^{j}\left(x,y\right)=\left|C_{S}^{j}\left(x,y\right)-\underset{\left(x^{\prime },y^{\prime }\right)\in p}{mean}\left(C_{S}^{j}\left(x+x^{\prime },y+y^{\prime }\right)\right)\right| \end{array}$$

Where *S* represents the two color multi-focus images *A* and *B* to be fused, *j* represents the decomposition scale, $C_{S}^{j}\left(x,y\right)$ is the low-frequency sub-band coefficient of the original image *S* on scale *j* at pixel (*x, y*), *P* is the range of the coefficient window, $a_{S}^{j}\left(x,y\right)$ is the activity level of $C_{S}^{j}\left(x,y\right)$ at pixel (*x, y*), and *mean*(·) represents mean filtering. Experiments show that the visual effect after image fusion is the most optimal when *P* uses 3×3 local windows.

#### Matching Level

The matching level between A and B pixels of two color multi-focus images can be measured by the color differences between them, which can be calculated with the quaternion rotation theory (Jin et al., 2013). As the color difference includes chromaticity and luminance, the formula for calculating the matching level is as follows:

(11) $$\begin{array}{l} m_{AB}^{j}\left(x,y\right)=t|Q\left(q_{1},q_{2}\right)|+\left(1-t\right)|I\left(q_{1},q_{2}\right)| \end{array}$$

In Equation (11), *q*_1_ = *r*_1_*i* + *g*_1_*j* + *b*_1_*k* and *q*_2_ = *r*_2_*i* + *g*_2_*j* + *b*_2_*k* are the pixels represented by quaternions in the color original images *A* and *B*, respectively. *Q*(*q*_1_, *q*_2_) and *I*(*q*_1_, *q*_2_) denote the differences in chromaticity and luminance, respectively, between *q*_1_ and *q*_2_, the weight *t* ∈ [0, 1] indicates the relative importance of chromaticity and luminance, and *j* represents the decomposition scale. According to the theory of quaternion rotation, the relationship between *q*_1_ and *q*_2_ can be expressed as $q_{\text{3}}=q_{1}+Rq_{2}R^{*}=r_{3}\cdot i+g_{3}\cdot j+b_{3}\cdot k$, *R* = *e*^*uπ*/2^, $u=\left(i+j+k\right)/\sqrt{3}$. If the chromaticity of *q*_1_ is similar to that of *q*_2_, *q*_3_ should be near the grayscale line *u*, and the chromaticity difference between *q*_1_ and *q*_2_ can be expressed by the following equation:

(12) $$\begin{array}{l} Q(q_{1},q_{2})\;=(r_{3}-(r_{3}+g_{3}+b_{3})/3)\cdot i+(g_{3}-(r_{3}+g_{3}\\ \;+b_{3})/3)\cdot j+(b_{3}-(r_{3}+g_{3}+b_{3})/3)\cdot k \end{array}$$

When *Q*(*q*_1_, *q*_2_) is small, the chromaticity of *q*_1_ and *q*_2_ are similar; when *Q*(*q*_1_, *q*_2_) = 0, *q*_1_ and *q*_2_ have the same chromaticity. The difference in luminance between *q*_1_ and *q*_2_ can be illustrated as:

(13) $$\begin{array}{l} I\left(q_{1},q_{2}\right)=\left(r_{1}-r_{2}\right)/3+\left(g_{1}-g_{2}\right)/3+\left(b_{1}-b_{2}\right)/3 \end{array}$$

According to Equations (11–13), the size of $m_{q_{1}q_{2}}^{j}$ is proportional to the color difference between *q*_1_ and *q*_2_. Therefore, the matching level between the two pixels can be measured by the size of the color difference.

#### Decision Plan

The decision value of the color image focus judgment is determined by the activity level and matching level of the local window. They are obtained by Equations (10, 11), respectively. The decision value is calculated by the following formula:

(14) $$\begin{array}{l} d^{j}\left(x,y\right)=\{\begin{array}{cc} 1, & if\;m_{AB}^{j}\left(x,y\right)>T\;and\;a_{A}^{j}\left(x,y\right)\geq a_{B}^{j}\left(x,y\right)\\ 0, & if\;m_{AB}^{j}\left(x,y\right)>T\;and\;a_{A}^{j}\left(x,y\right)<a_{B}^{j}\left(x,y\right)\\ \frac{1}{2}+\frac{1}{2}\left(\frac{1-T}{1-m_{AB}^{j}\left(x,y\right)}\right), & if\;m_{AB}^{j}\left(x,y\right)\leq T\;and\;a_{A}^{j}\left(x,y\right)\geq a_{B}^{j}\left(x,y\right)\\ \frac{1}{2}-\frac{1}{2}\left(\frac{1-T}{1-m_{AB}^{j}\left(x,y\right)}\right), & otherwise \end{array} \end{array}$$

According to the decision value *d*^*j*^(*x, y*), the fused low-frequency image can be obtained using $F_{L}^{j}\left(x,y\right)=d^{j}\left(x,y\right)*A_{L}^{j}\left(x,y\right)+\left(1-d^{j}\left(x,y\right)\right)*B_{L}^{j}\left(x,y\right)$, where $F_{L}^{j}\left(x,y\right)$ represents the low-frequency sub-image after the fusion of $A_{L}^{j}\left(x,y\right)$ and $B_{L}^{j}\left(x,y\right)$ at scale *j*. In Equation (14), *T* is the matching threshold between the pixel A and pixel B of a multi-focus image.

### High-Frequency Component Fusion Rules

In Equation (8), the first row of *S* represents low-frequency component of the original image, which carries the primary information from the image. The other rows *S*(2 : *mn*, :) of *S* denotes the high-frequency components of the original image, presenting the details of the image. According to the orthogonality of singular value decomposition, each component forms an orthogonal complement on the same scale. The direct sum of each component is:

(15) $$\begin{array}{l} I_{j}=I_{j+1}⊕\sum_{i=2}^{mn}S{\left(i,:\right)}_{j+1}\;\left(j=2,1,0\right) \end{array}$$

where *j* represents the decomposition scale; when *j* = 2, the highest decomposition layer is 3, *I*_3_ = *S*(1, :)_3_, and each component can be written as:

(16) $$\begin{array}{l} \{\begin{array}{l} I_{2}=S{\left(1,:\right)}_{3}⊕\sum_{i=2}^{mn}S{\left(i,:\right)}_{3}\;j=\text{2,}\\ I_{1}=I_{2}⊕\sum_{i=2}^{mn}S{\left(i,:\right)}_{2}\;j=\text{1,}\\ I_{0}=I_{1}⊕\sum_{i=2}^{mn}S{\left(i,:\right)}_{1}\;j=\text{0}, \end{array} \end{array}$$

The high-frequency sub-images of QMSVD reflect the detailed characteristics of the original image. Most of the fused methods operate in the feature domain of high-frequency components, without taking the influence of low frequency into account, compromising the fusion quality. To factor in the influence of low-frequency components in high-frequency component fusion, a local contrast fusion rule, which is applicable to both high-frequency and low-frequency regions, is proposed. After the original image is decomposed by QMSVD, the local contrast of the high-frequency and low-frequency components can be obtained by the following equation (Pu and Ni, 2000):

(17) $$C_{S_{j}}^{k}(x,y)=I_{S_{j}}^{H_{k}}(x,y)/I_{AB_{j}}^{L}(x,y),(S_{j}=A_{j}orB_{j})$$

In Equation (17), $I_{AB_{j}}^{L}$ represents the fusion component of the low-frequency sub-image of the original image *A* and *B* at scale *j*, and $I_{S_{j}}^{H_{k}}$ represents the *k-th* high-frequency component of the original image *S* at scale *j*. According to Equation (15), the high-frequency is not aliased with low-frequency components, and therefore the definition of the local contrast mirroring the high-frequency components is valid. The high-frequency sub-image fusion is defined as:

(18) $$\begin{array}{l} H_{F_{j}}^{k}\left(x,y\right)=\{\begin{array}{l} I_{A_{j}}^{H_{k}}\left(x,y\right),if\left|C_{A_{j}}^{k}\left(x,y\right)\right|\geq \left|C_{B_{j}}^{k}\left(x,y\right)\right|\\ I_{B_{j}}^{H_{k}}\left(x,y\right),otherwise \end{array} \end{array}$$

where $H_{F_{j}}^{k}\left(x,y\right)$ represents the *k*th high-frequency component of the fused image *F* at scale *j*.

### Multi-Focus Color Image Fusion Process

**Figure 2:**
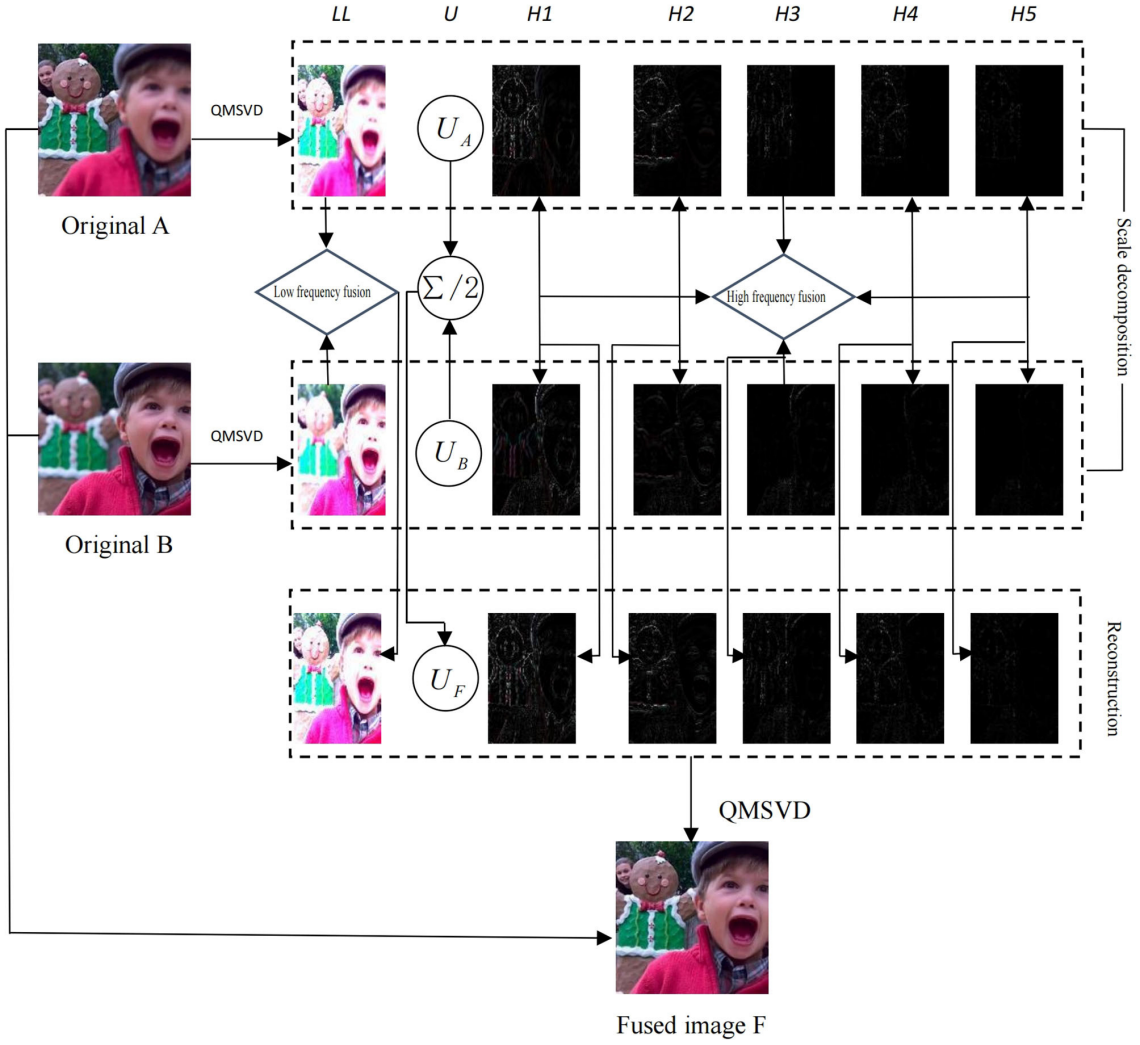
Fusion of sub-images by QMSVD with six channels. *LL* is the low-frequency component of the decomposed image, *H1–H5* are the high-frequency components of the decomposed image, *U*_*A*_ and *U*_*B*_ are the orthogonal matrices of the decomposed image, and *U*_*F*_ = (*U*_*A*_ + *U*_*B*_)/2.

Figure 2 shows the scheme of multi-focus color image fusion based on QMSVD with six channels, and the corresponding fusion process is as follows:

Step 1: Two original color multi-focus images A and B are decomposed by QMSVD. The low-frequency sub-image *A*_*L*_, *B*_*L*_ is represented by one channel and the high-frequency sub-images *A*_*H*_*i*__, *B*_*H*_*i*__ (*H*_*i*_ is the *i*th high-frequency channel) are represented by multiple channels. The orthogonal matrices *U*_*A*_ and *U*_*B*_, corresponding to singular values, are also obtained.
Step 2: The low-frequency sub-images *A*_*L*_, *B*_*L*_ are fused following low-frequency fusion rules, and the high-frequency sub-images *A*_*H*_*i*__, *B*_*H*_*i*__ are fused using high-frequency fusion rules.
Step 3: The orthogonal matrices *U*_*A*_ and *U*_*B*_ (obtained in Step 1) are fused. In the fusion of two images after QMSVD decomposition, the roles of *U*_*A*_ and *U*_*B*_ are identical, so the fusion rule for the orthogonal matrix is: *U*_*F*_ = (*U*_*A*_ + *U*_*B*_)/2.
Step 4: The final fusion image is obtained by inverse QMSVD transform of the fusion results in Step 2 and Step 3.

## Experimental Results and Discussion

In this study, color information richness (CCM) (Yuan et al., 2011), spatial frequency (SF), image contrast metric (ICM) (Yuan et al., 2011), and edge information retention (QAB/F) (Liu et al., 2012) are utilized to evaluate the multi-focus color fusion image objectively, and to verify the effectiveness of the algorithm. The CCM index value is determined by the color chromaticity and color difference gradient of the fused image. The SF index reflects the clarity of the image details. The ICM index is composed of the grayscale contrast and color contrast of the fused image, with the value denoting the contrast in the fused image. The QAB/F index implies how much information about edge and structure from the original image is retained in the fused image. For the above evaluation indicators, a larger evaluation value suggests a better fusion result.

The proposed QMSVD color image fusion method is compared with five typical multi-focus image fusion methods, which fall into the category of the multi-resolution singular value decomposition fusion method (MSVD) (Naidu, 2011), the Multi-scale weighted gradient-based fusion method (MWGF) (Zhou et al., 2014), the boosted random walks-based fusion method (RWTS) (Ma et al., 2019), the guided fifilter-based fusion method (GFDF) (Qiu et al., 2019), the deep CNN fusion method (CNN) (Liu et al., 2017). Among them, the MSVD, MWGF, RWTS and GFDF are traditional image fusion methods. The CNN is a recently proposed image fusion method based on deep learning. In Liu et al. (2017), Liu chooses the Siamese as the CNN model, and the network has three convolutional layers and one max-pooling layer. The training sample is a high-quality natural image of 50,000 from the ImageNet dataset, and input patch size is set at 16 × 16. The Matlab implementation of the above five fusion methods are all obtained online, and the parameters are the default values given in the literature. The original multi-focus images used in the experiment are obtained from multiple image datasets. The four images (A), (B), (D), (E) in **Figure 4** and the one image (I) in **Figure 6** are obtained from the Lytro dataset (Nejati et al., 2015). The Six images (A)–(F) in **Figure 6** are obtained from the Slavica dataset (Slavica, 2011). The one image (C) in **Figure 4** and the two images (G) and (H) in **Figure 6** are obtained from the Saeedi dataset (Saeedi and Faez, 2015). The one image (J) in **Figure 6** is obtained from the Bavirisetti dataset (Bavirisetti). In this paper, five groups of color images with rich colors are selected in the image datasets Lytro and Saeedi, and they are used in the comparison experiment. In addition, 10 groups multi-focus images commonly used in other related papers as the experimental data are used in the comparison experiment, and they have different sizes and characteristics.

In the experimental process, firstly, the experimental parameters of the algorithm set prior to the experiment. Secondly, the fusion results achieved using the proposed algorithm and the other algorithms are presented and compared.

### Selection of Experimental Parameters

Multi-scale singular value decomposition of color images is conducted through multiple independent layers and channels. Image decomposition generally divides the image into three layers. Channel decomposition usually divides the image into four-channel, six-channel, eight-channel, and nine-channel. Channel decomposition is illuminated in Equation (9). The result of image fusion is also affected related to the size of the local window P, and the typical size is 3×3 or 5×5. The experimental comparison suggests, the 5×5 local window exceeds the size of the important feature of the image, which undermines the judgment of the local window activity. Therefore, in this paper, we set a local window size at *P* = *3* × *3*. As can be observed from Equation (11), the weight *t* ∈ [0, 1] indicates the relative importance of chromaticity and luminance, with t positively related to chromaticity. In Equation (14), T represents the matching threshold of the matching level between the pixels of the two color multi-focus images to be fused, and the value of T directly affects the decision value *d*(*x, y*) of low-frequency fusion. The parameters discussed above ultimately determine the effect of image fusion.

**Figure 3:**
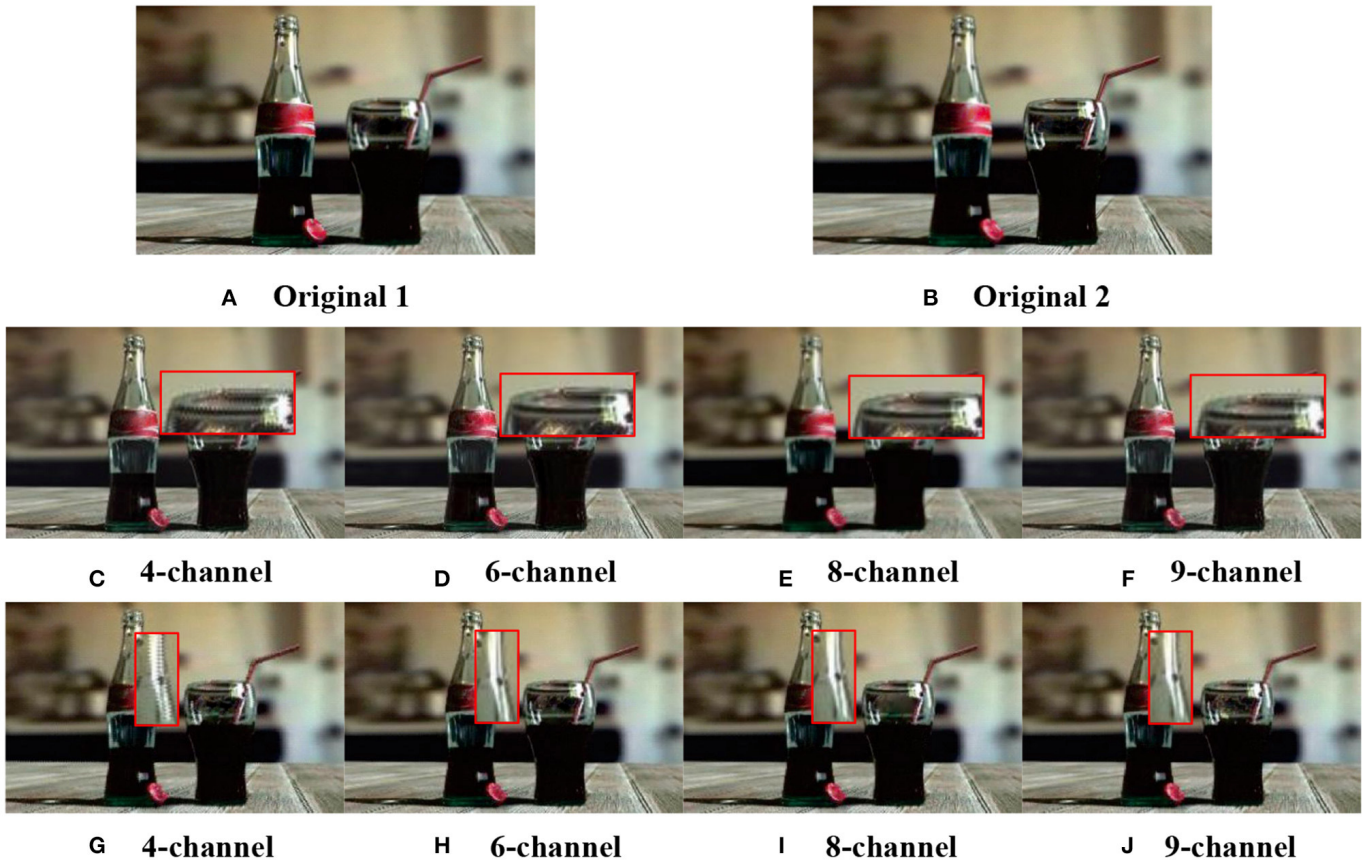
Tested multi-focus color image. **(A,B)** are original images. The parameters are selected: in **(C–F)**, layer = 2, *P* = 3, *t* = 0.9, *T* = 0.01; in **(G–J)**, layer = 2, *P* = 3, *t* = 0.9, *T* = 0.03; with four, six, eight, and nine decomposition channels.

We set different parameter values, conducted repeated comparative experiments, and used two objective indices spatial frequency (SF) and color colorfulness metric (CCM) (Yuan et al., 2011) to evaluate Figure 3. As Table 1 reveals, the SF value decreases as the number of channels increases, the larger the number of channels the smoother the image after multi-scale singular value decomposition, and the lower the spatial frequency. The maximum value of CCM occurs when *t* = 0.9. According to Equation (11), value *t* indicates the importance of chromaticity. The analysis shows that the algorithm proposed in this paper is feasible. From further analysis in Table 1, the preliminary parameters could be obtained: *P* = *3, t* = *0.9, T* = *0.01*, and *P* = *3, t* = *0.9, T* = *0.03*, with six and eight decomposition channels.

**Table 1:** Selection of initial parameters (1).

**Parameters**	**Decompose**	**Spatial frequency (SF)**				**Color colorfulness metric (CCM)**			
		**4-channel**	**6-channel**	**8-channel**	**9-channel**	**4-channel**	**6-channel**	**8-channel**	**9-channel**
*t* = 0.8, *T* = 0.01	1-layer	27.7544	27.1201	27.0089	24.0205	17.1906	17.2659	17.3272	17.1945
	2-layer	27.7476	27.1008	26.9451	23.9930	17.2387	17.3145	17.3351	17.1784
	3-layer	27.7046	27.0782	26.9962	24.0224	17.2168	17.3000	17.2959	17.1949
*t* = 0.8, *T* = 0.02	1-layer	27.7530	27.1597	27.0169	24.0233	17.1766	17.2540	17.3045	17.1713
	2-layer	27.7565	27.0789	26.9332	23.9857	17.1433	17.2997	17.3361	17.1717
	3-layer	27.7017	27.0856	26.9821	23.9763	17.1764	17.2824	17.2891	17.1752
*t* = 0.8, *T* = 0.03	1-layer	27.7701	27.1563	27.0112	24.0128	17.1863	17.2646	17.3160	17.1813
	2-layer	27.7474	27.0726	26.9467	24.0115	17.1571	17.2842	17.3379	17.1754
	3-layer	27.6838	27.0666	26.9722	23.9911	17.1753	17.2689	17.2997	17.1662
*t* = 0.9, *T* = 0.01	1-layer	27.7659	27.1194	**27.0250**	24.0425	17.1872	17.2721	17.3282	17.2052
	2-layer	27.7655	27.0759	26.9507	24.0050	17.2252	**17.3203**	17.3368	17.1696
	3-layer	27.7285	27.0611	26.9984	24.0227	17.2239	17.3126	**17.3529**	17.1971
*t* = 0.9, *T* = 0.02	1-layer	27.7560	27.1401	27.0168	24.0097	17.1729	17.2506	17.2962	17.1755
	2-layer	27.7343	27.0708	26.9335	23.9877	17.1538	17.3002	17.3354	17.1735
	3-layer	27.6954	27.0692	26.9641	23.9779	17.1793	17.2707	17.2944	17.1755
*t* = 0.9, *T* = 0.03	1-layer	27.7818	**27.1624**	27.0196	23.9995	17.1861	17.2599	17.3134	17.1780
	2-layer	27.7563	27.0767	26.9629	24.0269	17.1555	17.2871	17.3332	17.1750
	3-layer	27.7052	27.0612	26.9701	24.0058	17.1757	17.2689	17.3034	17.1634

*The numbers in bold indicate the maximum value obtained with different objective evaluation indicators*.

Figure 3 demonstrates the results obtained in the second decomposition layer using the preliminary parameters analyzed above. Obviously, the fusion image based on four channel decomposition has the worst visual effect, and the edge of detail appears zigzag distortion, which results from the block effect caused by small channel decomposition. Artifacts emerge at the edge of fused image obtained through nine- channel decomposition. This due to the large channel decomposition which lead to blurring of the fused image. Fused images obtained through six-channel and eight-channel decomposition have similar effects and the best quality. Judging from the Table 1, it can be concluded that the subjective visual effects are consistent with the objective evaluation values. In other words, the objective evaluation value is positively proportional to the subjective visual effect.

From the analysis above, the fusion effects of the six-channel and eight-channel decomposition are superior to those of the four-channel or nine-channel decomposition. Further analysis from Table 2 reveals that the overall results of SF and CCM with six channels are better than those with eight channels, therefore, we finally adopt the six-channel decomposition approach. According to Table 1, during the six-channel decomposition, when *P* = 3, *t* = 0.9, *T* = 0.03, and layer = 1, the maximum SF value is 27.1624, and when layer = 2, the maximum CCM value is 17.2871. To optimize the result of multi-focus color image fusion, we take into account importance of color evaluation index CCM in color image fusion, and take the six-channel decomposition approach, and set *P* = 3, *t* = 0.9, *T* = 0.03, and layer = 2.

**Table 2:** Selection of initial parameters (2).

**Channel**	**Metrics**	***t* = 0.8, *T* = 0.01**	***t* = 0.8, *T* = 0.02**	***t* = 0.8, *T* = 0.03**	***t* = 0.9, *T* = 0.01**	***t* = 0.9, *T* = 0.02**	***t* = 0.9, *T* = 0.03**	**Total**
6-channel	SF	81.2991	81.3242	81.2955	81.2564	81.2801	81.3003	798.8322
	CCM	51.8804	51.8361	51.8177	51.905	51.8215	51.8159	
8-channel	SF	51.9582	51.9297	51.9536	52.0179	51.926	51.95	797.389
	CCM	80.9502	80.9322	80.9301	80.9741	80.9144	80.9526	

### Subjective Evaluation

To verify the performance of the proposed method of multi-focus color image fusion in terms of visual perception, 15 groups of multi-focus color images are selected for our experiment. Five groups come from the multi-focus image data set “Lytro,” while the other 10 groups are widely used in multi-focus image fusion. Meanwhile, the proposed fusion method is compared with five typical multi-focus image fusion methods, which are the MSVD, MWGF, RWTS, GFDF and CNN.

**Figure 4:**
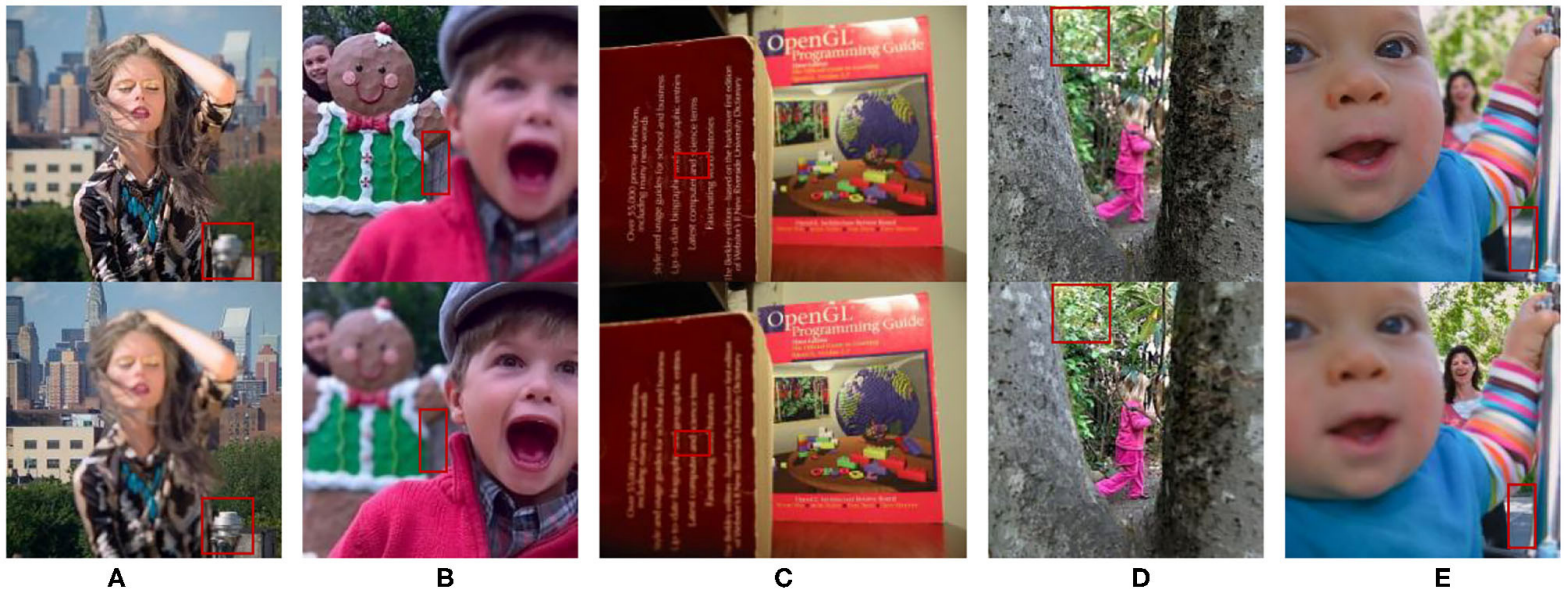
Five groups of multi-focus color original images. Red frames are the area that need to be compared in image fusion. **(A)** Woman, **(B)** Child, **(C)** Book, **(D)** Girl, and **(E)** Baby. The four images **(A,B,D,E)** from Lytro dataset, the image **(C)** from Saeedi dataset.

**Figure 5:**
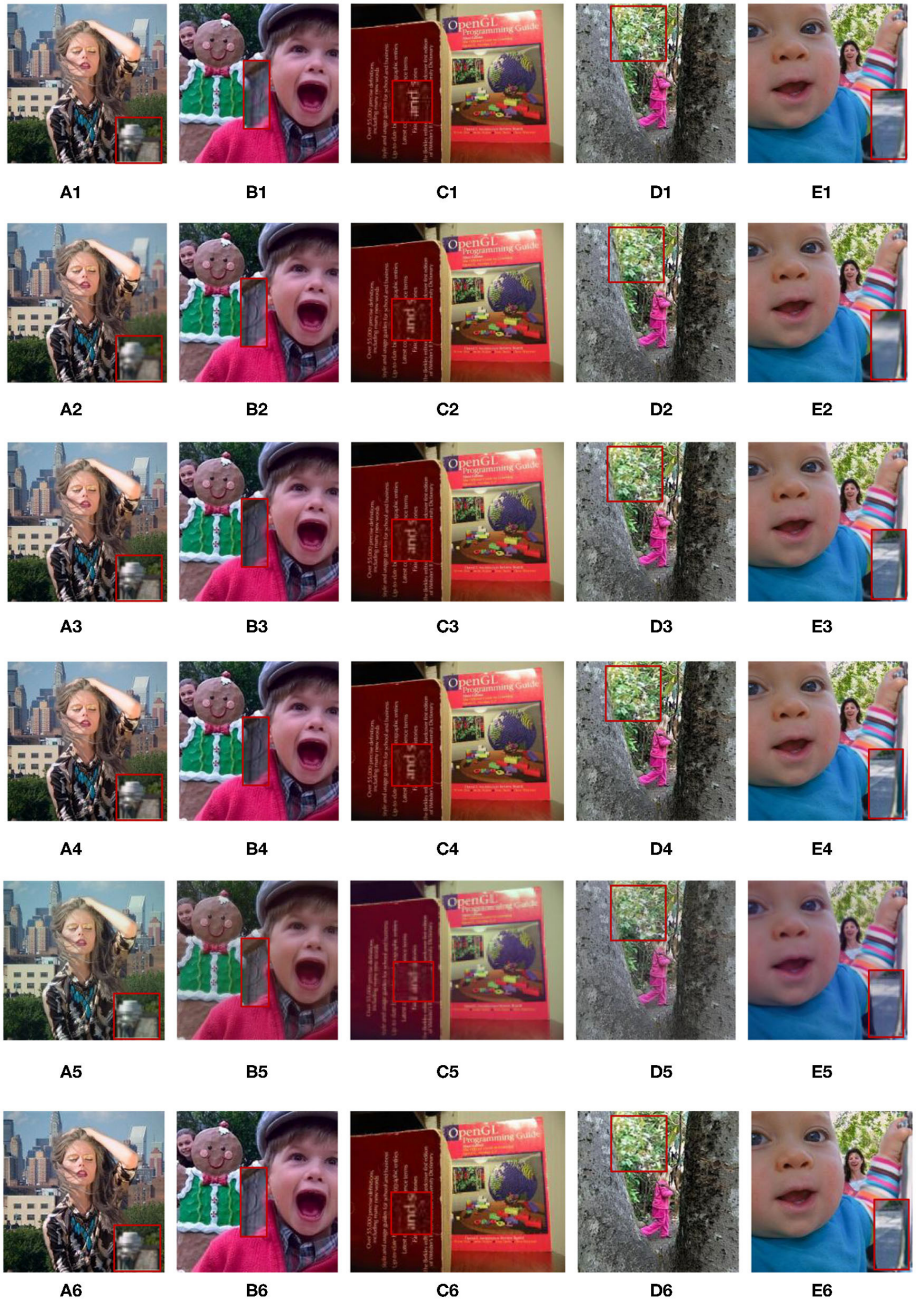
Corresponding to the fusion results of the five original images in Figure 4. **A(1)–E(1)** are the fusion images obtained by the GFDF method. **A(2)–E(2)** are the fusion images obtained by the MWGF method. **A(3)–E(3)** are the fusion images obtained by the CNN method. **A(4)–E(4)** are the fusion images obtained by the RWTS method. **A(5)–E(5)** are the fusion images obtained by the MSVD method. **A(6)–E(6)** are the fusion images obtained by the QMSVD method.

In Figure 4, we select five groups images from the multi-focus data set “lytro” for experiments. They have rich colors, which are also the experimental data used in the five comparison algorithms. The areas in each image that need to be compared are marked with a red frame. Figure 5 is the fusion result corresponding to the five original images in Figure 4. For better comparison, the red frame areas in the fusion image are enlarged.

Group A(1)–A(6) show the images of the “woman” with the size of 208 × 208 and the fused image obtained by 6 different fusion methods. The comparison of red framed areas suggest the QMSVD, RWTS, MSVD, and GFDF have the best visual clarity, followed by CNN, and MDGF is the most blurry. A further comparison shows that in the fused image obtained by the MSVD, the red framed region and the image of “woman” have obvious color distortion.

Group B(1)–B(6) show the images of the “child” with the size of 256 × 256 and the fused image obtained by six different fusion methods. The comparison of the red framed areas demonstrates that the QMSVD and MWGF have the best visual clarity, and GFDF is the fuzziest. A further comparison shows that in the fusion image obtained by the MSVD, the face brightness of “child” is the lowest.

Group C(1)–C(6) show the images of the “book” with the size of 320 × 240 and the fused image obtained by six different fusion methods. Comparing the English letters in the red frame area of each image. From a visual point of view, the MSVD-based method is the most blurry, and fusion effects achieved by the other methods are similar.

Group D(1)–D(6) show the images of the “girl” with the size of 300 × 300 and the fused image obtained by six different fusion methods. Comparing the leaves in the red frame area of each image, the QMSVD and the RWTS can produce the best fusion image effect, and the color is close to the original image.

Group E(1)–E(6) show the images of the “baby” with the size of 360 × 360 and the fused image obtained by 6 different fusion methods. The comparison illustrates that the QMSVD, CNN, and RWTS obtain the best fusion image effects, followed by the GFDF and MSVD, and the MWGF lags behind.

**Figure 6:**
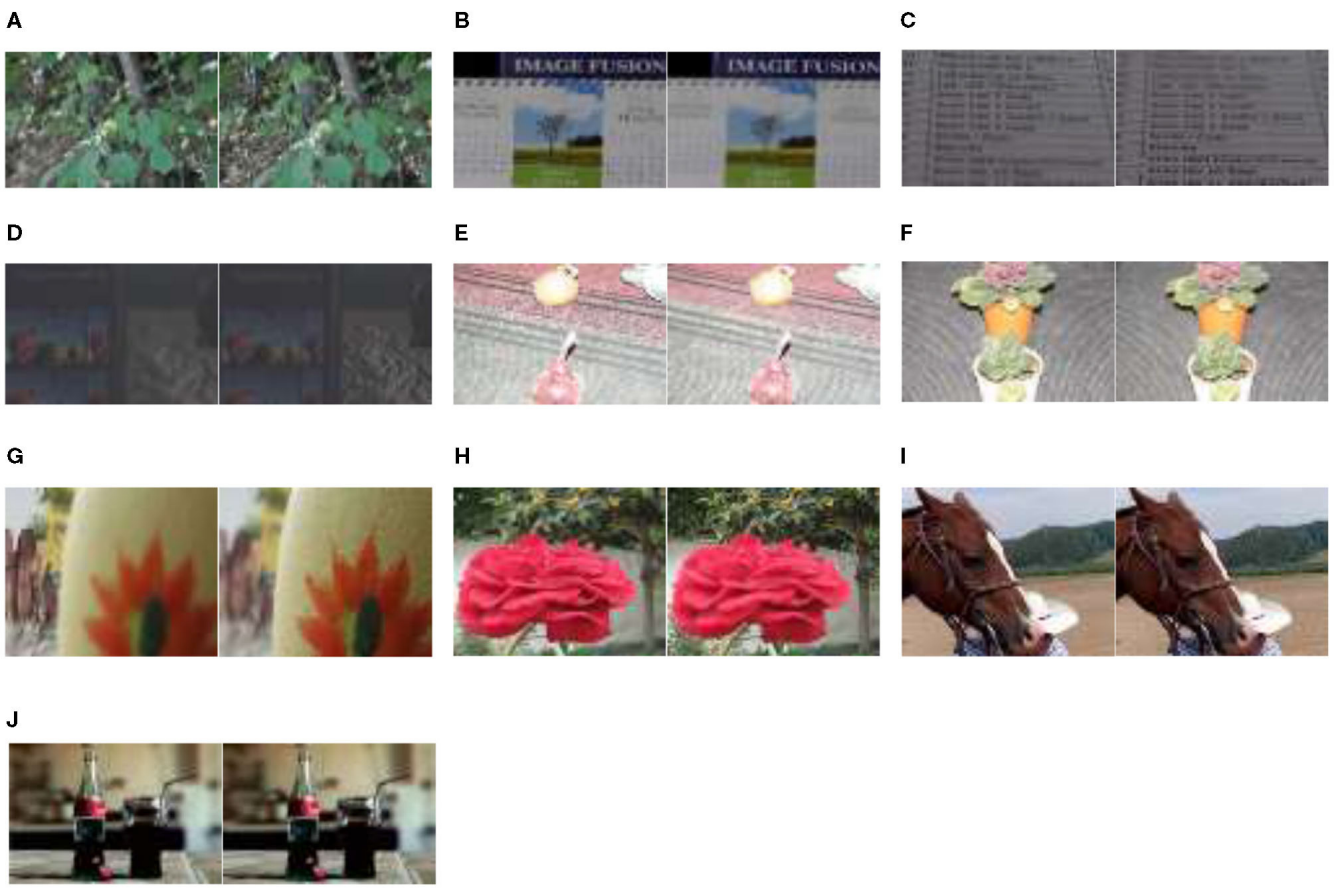
Ten groups of multi-focus color images. **(A)** Size of 267×171, **(B)** size of 267×175, **(C)** size of 267×177, **(D)** size of 267×177, **(E)** size of 267×174, **(F)** size of 320×200, **(G)** size of 267×174, **(H)** size of 390×260, **(I)** size of 222×148, and **(J)** size of 360×360. The six images **(A–F)** from Slavica dataset, the two images **(G,H)** from Saeedi dataset, the image **(I)** from Lytro dataset and the image **(J)** from Bavirisetti dataset.

**Figure 7:**
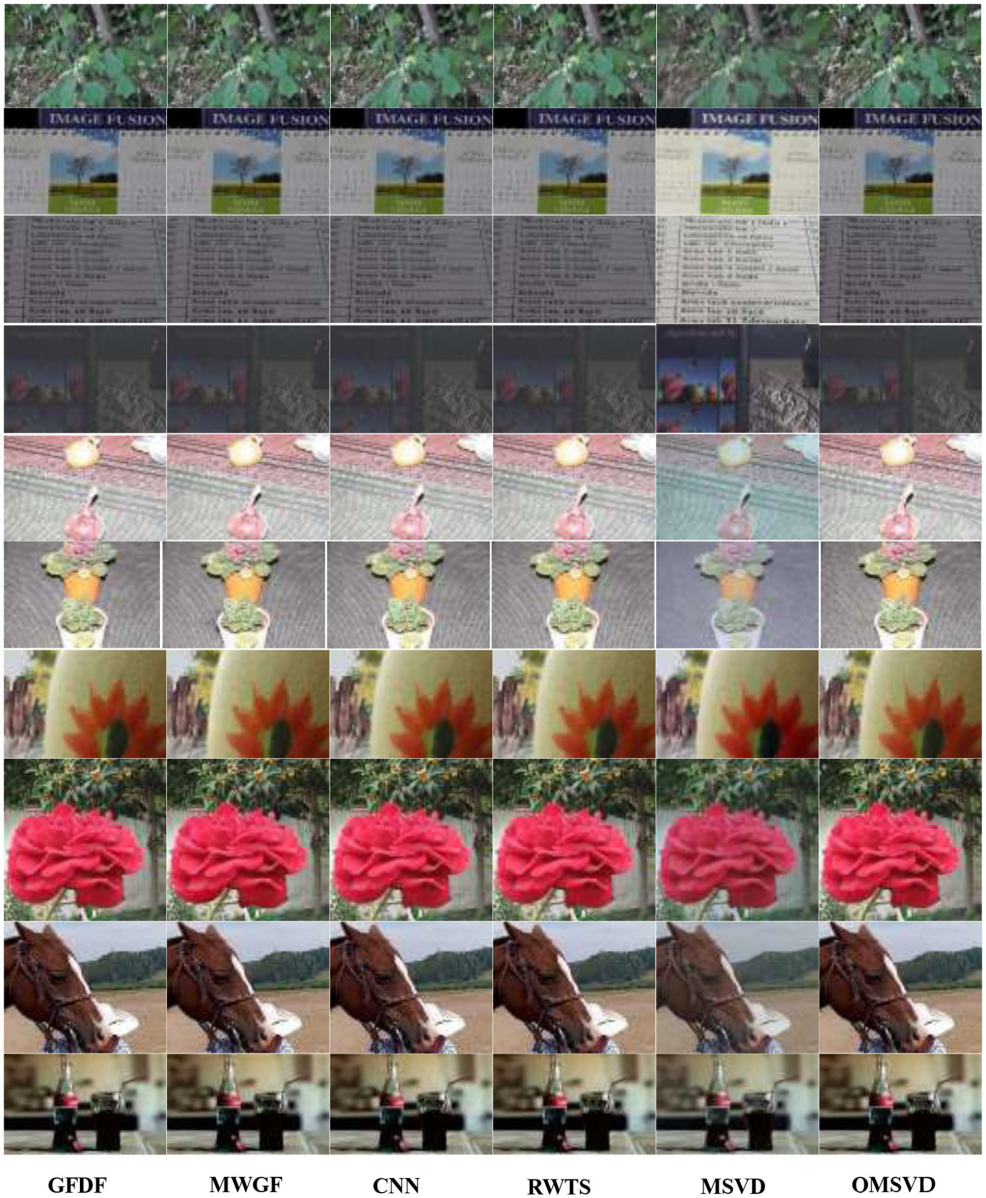
Ten groups of multi-focus color fusion images.

**Figure 8:**
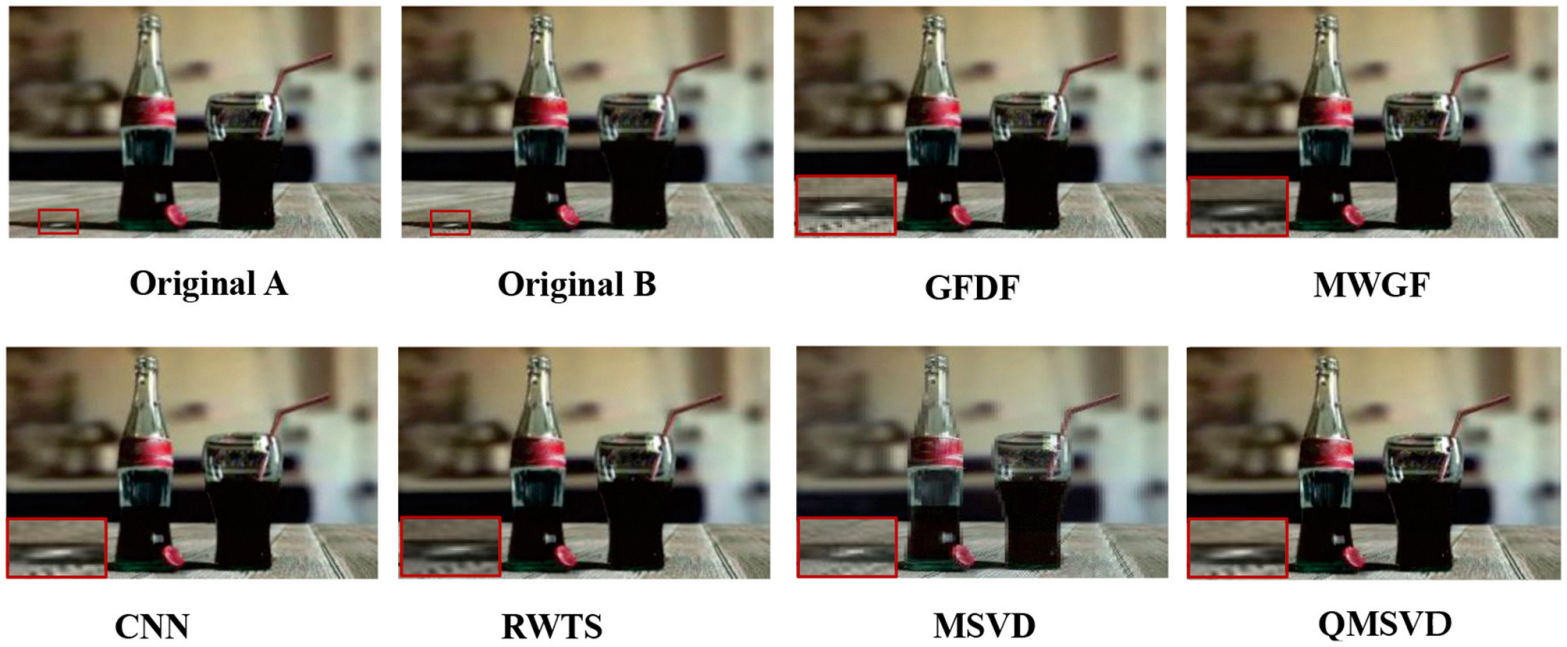
“Coke Bottle” fusion images obtained by six different fusion methods.

**Figure 9:**
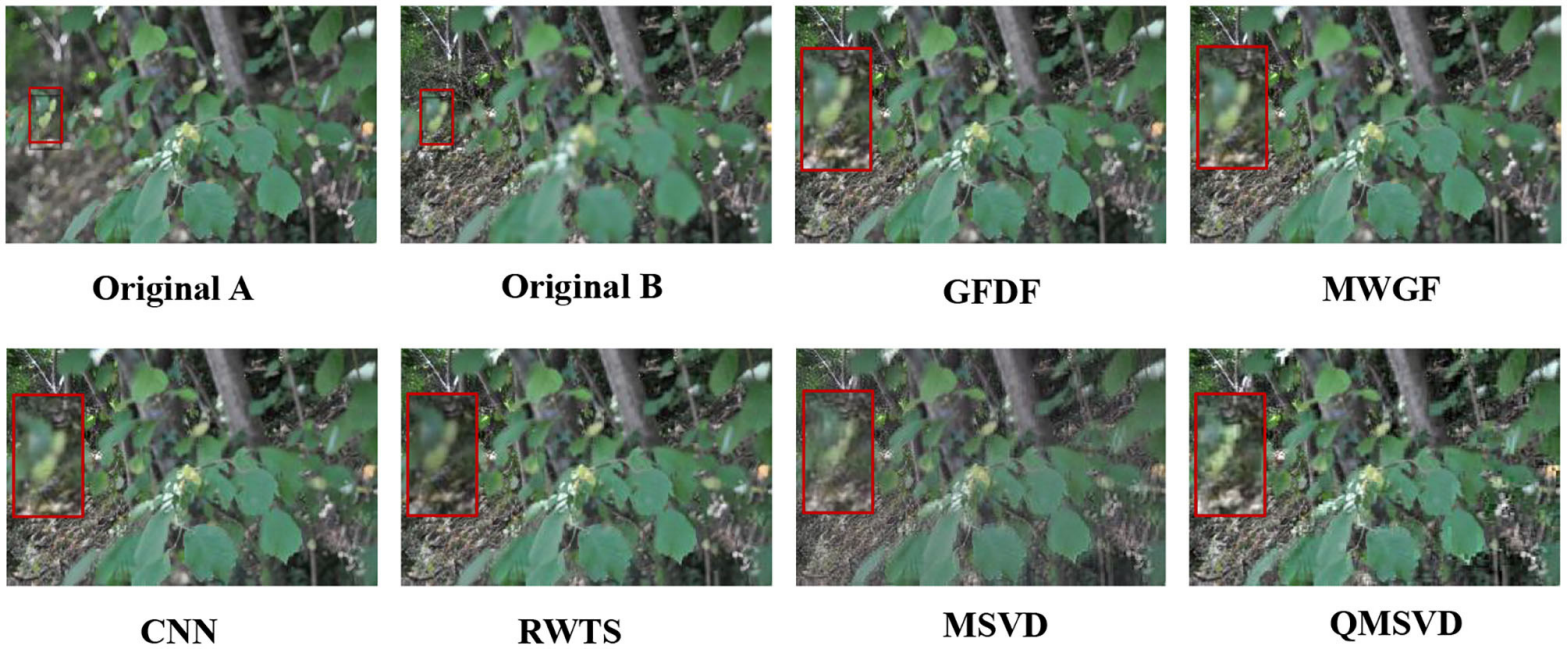
“Forest” fusion images obtained by six different fusion methods.

To further prove the effectiveness of the QMSVD method for multi-focus color image fusion, the 10 groups of original images are given in Figure 6. In Figure 7, the fused image obtained by six different fusion methods are shown. In Figures 8, 9, we compare two groups of images in detail.

In Figure 8, the original image of “Coke Bottle” with a size of 320 × 200 and the fused image obtained by six different fusion methods are shown. Compare the bright spots in the red frame area of each image, QMSVD, CNN, and GFDF achieve better clarity, followed by MWGF and RWTS, and MSVD is the most ambiguous.

In Figure 9, the original image of “Forest” with a size of 267 × 171 and the fused image obtained by six different fusion methods are shown. Compare the brightness of leaves in the red frame area of each image, QMSVD, superior to other methods, obtains the best fusion image effect.

In general, the QMSVD method combines the advantages of quaternions and multi-scale decomposition in color multi-focus image fusion. The benefit is that quaternions can represent and process different color channels of a color image as a whole, producing the fused multi-focus image with high fidelity. Multi-scale decomposition methods decompose the image into low-frequency and high-frequency components at different levels. In this way, the decomposed images can be fused accurately at different components, scales, and levels, which renders the fused color multi-focus image with high definition and contrast, and good visual effects.

### Objective Evaluation

We proposed the method for multi-focus color image fusion. We classify experimental images in two categories. One type is multi-focus color pictures with rich color information, and their objective evaluation metrics of different methods are presented in Table 3. The other type is commonly used multi-focus color images. We have selected two groups, and their objective evaluation metrics of different methods are counted in Table 4. Table 5 is the average objective evaluation metrics of different methods on 15 groups color images. The analysis of Tables 3–5 shows that the average values of the 15 groups using CCM and ICM indicators of the QMSVD algorithm are significantly higher than those of other fusion algorithms. This also shows that the fused image has a high definition and rich color, which is consistent with the visual performance of the fused image in the subjective evaluation. Of all the fusion algorithms, the CCM index of the QMSVD algorithm ranks first. For the QAB/F indicator, the QMSVD algorithm performs worse than other algorithms in preserving edge and structure information. In general, the QMSVD method achieves the best results on the CCM indicator and performs well on the ICM and SF indicators. This shows that the QMSVD method is effective, and the fused image has a high definition, rich color, less information loss, and good overall visual effects.

**Table 3:** Objective evaluation values of multi-focus color images.

**Image**	**Metrics**	**GFDF**	**MWGF**	**RWTS**	**CNN**	**MSVD**	**QMSVD**	**Rank**
“Woman”	CCM	19.8116	19.6341	19.8065	19.8219	19.2142	**19.9050**	1
	ICM	0.5448	**0.5555**	0.5447	0.5451	0.5463	0.5538	2
	SF	30.2661	29.4158	30.3256	29.8675	27.3840	**30.8316**	1
	QAB/F	0.6845	0.6656	0.6847	**0.6866**	0.6523	0.6692	4
“Child”	CCM	26.6408	26.5463	26.6100	26.5925	25.5832	**26.7334**	1
	ICM	0.4910	0.4913	0.4912	0.4915	0.3638	**0.4988**	1
	SF	25.1688	24.9778	24.8987	24.6005	18.9458	**25.4489**	1
	QAB/F	0.6240	0.6202	**0.6251**	0.6248	0.5054	0.5955	5
“Book”	CCM	28.7924	28.7851	28.7922	28.7846	26.9576	**28.9861**	1
	ICM	0.4582	0.4578	0.4578	0.4578	0.3506	**0.4610**	1
	SF	35.3490	**35.5172**	35.3293	35.2197	17.5239	33.9891	5
	QAB/F	0.6832	0.6814	0.6848	**0.6853**	0.3768	0.5944	5
“Girl”	CCM	20.5994	20.5039	20.5796	20.5546	17.5183	**20.6437**	1
	ICM	0.5311	0.5313	0.5312	0.5317	0.4446	**0.5340**	1
	SF	48.7194	48.5660	48.3491	47.8869	35.3703	**48.8196**	1
	QAB/F	0.6992	0.6943	0.7015	**0.7023**	0.6260	0.6854	5
“Baby”	CCM	24.9107	24.8468	24.9080	24.9040	16.6895	**24.9657**	1
	ICM	0.5161	0.5377	0.5161	0.5162	0.4229	**0.5739**	1
	SF	**19.4409**	19.1723	19.3729	19.2464	13.3334	19.3610	3
	QAB/F	0.6682	0.6599	0.6701	**0.6712**	0.5066	0.6479	5

*The numbers in bold indicate the maximum value obtained with different objective evaluation indicators*.

**Table 4:** Objective evaluation metrics of multi-focus color images in Figures 8, 9.

**Image**	**Metrics**	**GFDF**	**MWGF**	**RWTS**	**CNN**	**MSVD**	**QMSVD**	**Rank**
“Coke Bottle”	CCM	17.2691	17.2181	17.2865	17.2782	**15.1438**	**17.2871**	1
	ICM	0.5521	**0.5523**	0.5521	0.5521	0.4400	0.5508	2
	SF	**27.5118**	27.0422	27.4867	27.4254	19.1469	27.0767	4
	QAB/F	0.7609	0.7446	0.7609	**0.7613**	0.4820	0.7563	4
“Forest”	CCM	21.2723	21.2740	21.2442	21.2616	20.7242	**21.5211**	1
	ICM	0.4493	0.4496	0.4503	0.4495	0.4346	**0.5120**	1
	SF	26.5008	26.4351	26.6436	26.3499	23.4413	**29.6777**	1
	QAB/F	**0.6232**	0.6229	0.6188	0.6171	0.4182	0.4626	5

*The numbers in bold indicate the maximum value obtained with different objective evaluation indicators*.

**Table 5:** Average objective evaluation metrics of different methods on 15 groups color images.

**Image**	**Metrics**	**GFDF**	**MWGF**	**RWTS**	**CNN**	**MSVD**	**QMSVD**	**Rank**
**15 groups color images**	**CCM**	20.0358	19.9940	20.0308	20.0249	20.5252	**21.4558**	1
	**ICM**	0.4606	0.4641	0.4599	0.4581	0.3558	**0.4763**	1
	**SF**	**28.4214**	28.2365	28.3956	28.1854	23.6767	28.3095	3
	**QAB/F**	**0.6821**	0.6713	0.6820	0.6818	0.4619	0.6030	5

*The numbers in bold indicate the maximum value obtained with different objective evaluation indicators*.

## Conclusion

In this paper, a multi-focus color image fusion algorithm based on quaternion multi-scale singular value decomposition is proposed. In the algorithm, the color multi-focus image, represented by quaternions, undergoes multi-scale decomposition as a whole, avoiding the loss of color information caused by the multi-scale decomposition of each color channel separately. In addition, the algorithm can fuse the information of the decomposed image accurately in different components, scales, and levels. To verify the effectiveness of the algorithm, it has been analyzed qualitatively and quantitatively, and compared with the classical multi-scale decomposition fusion algorithm and fusion algorithms proposed in the latest literature. The experimental results show that the fusion result of this method reports great enhancement in the subjective visual effects. It also performs well in objective evaluation indices, particularly the CCM index of color information richness of the fused image. Because the algorithm proposed in this paper is based on multi-focus color images represented by quaternion, it takes more time to process the multi-scale decomposition of the images. Further research needs to be done to improve the efficiency of the algorithm and ensure the quality of image fusion. Regarding the setting of algorithm parameters, it is mainly based on empirical values, such as the selection of the number of channels, the selection of local window size, etc. In the future, the adaptive selection of parameters is also the focus of our future research. Additionally, the color images are not represented by the complete quaternion components, but by pure quaternion in image fusion. How to exploit the real part information of quaternion in color image processing will be our focus in the future study.

## Data Availability Statement

Publicly available datasets were analyzed in this study. This data can be found at: https://dsp.etfbl.net/mif/; https://mansournejati.ece.iut.ac.ir/content/lytro-multi-focus-dataset.

## Author Contributions

HW, XT, and BX conceived this research. HW and BX designed the algorithm. HW performed the computer simulations and wrote the original draft. HW and ZZ analyzed the data. WL and XT revised and edited the manuscript. All authors confirmed the submitted version.

## Conflict of Interest

The authors declare that the research was conducted in the absence of any commercial or financial relationships that could be construed as a potential conflict of interest.
